# A novel chimeric RNA RPGR-EEF1A1 enhances autophagy by interaction with the small GTPase RAB37 in a GTP-dependent manner

**DOI:** 10.1016/j.gendis.2026.102063

**Published:** 2026-01-31

**Authors:** Cong Li, Anqi Zhu, Wenhui Shi, Yifei Wang, Rongjia Zhou, Yanghua Tian, Zhimin Zhai, Qiang Hong

**Affiliations:** Department of Hematology, The Second Affiliated Hospital of Anhui Medical University, Hefei, Anhui 230601, China; School of Basic Medical Sciences, Anhui Medical University, Hefei, Anhui 230032, China; Hubei Key Laboratory of Cell Homeostasis, College of Life Sciences, Wuhan University, Wuhan, Hubei 430072, China; Department of Neurology, The Second Affiliated Hospital of Anhui Medical University, Hefei, Anhui 230601, China; Department of Hematology, The Second Affiliated Hospital of Anhui Medical University, Hefei, Anhui 230601, China; School of Basic Medical Sciences, Anhui Medical University, Hefei, Anhui 230032, China

Pre-mRNA splicing is an important event in the generation of mature mRNA across eukaryotes. *Trans*-splicing is a type of RNA splicing, in which two separate pre-mRNA molecules join to form a chimeric non-co-linear RNA that can generate novel proteins, noncoding or regulatory RNAs.^1^ These novel RNAs not only increase the complexity of the proteins but also provide new regulatory mechanisms for various biological processes. It is well known that *trans*-splicing is associated with a broad range of physiological and pathological processes, such as cell growth and cancer, gene expression regulation, and signal transduction. The chimeric RNAs generated by *trans*-splicing have the potential application value in treating human genetic diseases, such as skin disease, hemophilia A, and muscular dystrophy. Thus, it is important to identify novel *trans*-spliced transcripts in eukaryotes. In the present study, we identified a novel *trans*-spliced transcript *Rpgr-Eef1a1* in mice. We also found that RPGR-EEF1A1 interacted with RAB37 and stimulated the GDP-GTP exchange, ultimately promoting autophagy.

Autophagy is a widely conserved catabolic process that is essential for maintaining cellular homeostasis. Dysregulation of autophagy has been implicated in a variety of human diseases, including immune disorders, neurodegenerative diseases, cancers, and metabolic disorders. A fundamental question related to autophagy is how phagosomes mature into double-membrane autophagosome vesicles. Our previous study demonstrated that the small GTPase RAB37 functions as an organizer for autophagosome biogenesis via interaction with ATG5.^2^ However, GTPases need specific regulators for temporal control of the initiation of GTPase activation, and guanine nucleotide exchange factors (GEFs) can activate GTPases by stimulating the exchange of GDP to GTP. Thus, our further study revealed that a guanine nucleotide exchange factor, RPGR, can activate RAB37 GDP-GTP exchange and then coordinate retinal homeostasis by regulating autophagy.^3^ However, the regulatory relationship between *trans*-splicing molecules and autophagy has not been reported.

To characterize the *trans*-splicing events in mice, thirteen mouse tissues were used for screened through genome-wide analysis (Fig. S1). A *trans*-spliced transcript *Rpgr-Eef1a1* was identified, which occurred between retinitis pigmentosa GTPase regulator (*Rpgr*) on chromosomes X and eukaryotic translation elongation factor 1 alpha 1 (*Eef1a1*) on chromosomes 9. The splicing site overlapped between the short identical regions “GTAACAACTG” on both pre-mRNAs. The spliced molecule *Rpgr-Eef1a1* had an ORF with stop codon “TGA” at the junction site (Fig. 1A and B; Fig. S2A). To exclude chimera from chromosome translocation or artificial chimera from reverse transcriptases (RTases), two kinds of RTases (M-MLV and AMV) with different error rates and genomic DNA were used as controls. The results showed that *Rpgr-Eef1a1* could not be amplified from genomic DNA of mice, but could be PCR amplified from cDNAs transcribed by both RTases in retina (Fig. S2B and S2C). In addition, RPGR-EEF1A1 had obvious co-localization with its parental RPGR and EEF1A1 in the cytoplasm (Fig. S2D). *Rpgr-Eef1a1* was mainly expressed in the retina and ovary, and its parental molecules *Rpgr* and *Eef1a1* were ubiquitously expressed in the retina and ovary (Fig. S2E). Meanwhile, quantitative reverse transcription PCR was used to determine the relative expression levels of *Rpgr*, *Eef1a1*, and *Rpgr-Eef1a1*. The results showed that the expression of *Rpgr* was significantly higher than that of *Eef1a1* and *Rpgr-Eef1a1* in mouse retina and ovary (Fig. 1C). Further Hi-C analysis of the spatial organization of the mouse genome indicated that the chromatin region harboring *Rpgr* on chromosome X could interact with the region of *Eef1a1* on chromosome 9 (Fig. S2F and S2G). The Hi-C structure provided 3D chromatin contact of spatial organization for the *trans*-splicing.

**Figure 1:**
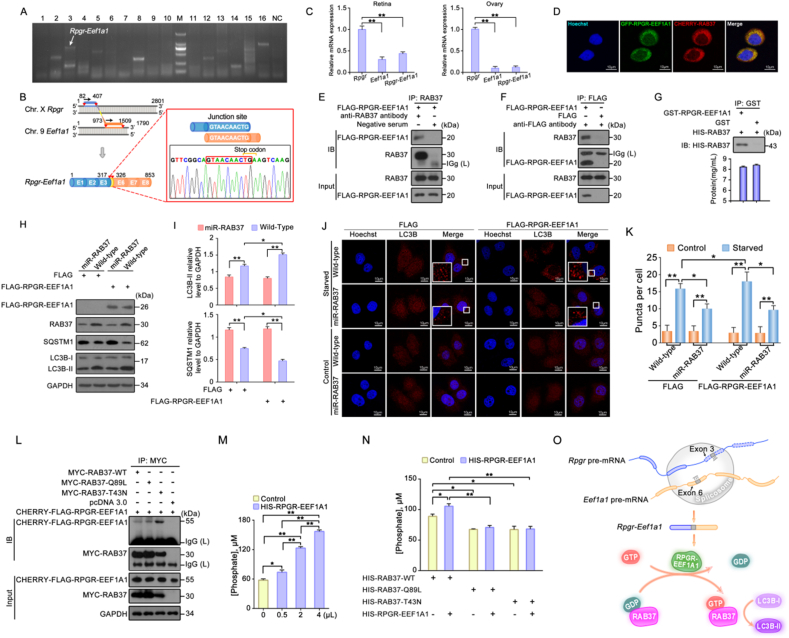
A novel chimeric RNA RPGR-EEF1A1 enhances autophagy by interaction with the small GTPase RAB37 in a GTP-dependent manner. **(A)** The candidate chimeric RNAs were verified by reverse transcription PCR. N.C.: no template control. DNA marker: 100, 250, 500, 750, 1000, and 2000 bp. **(B)***Trans*-splicing event of *Rpgr-Eef1a1*. Pre-mRNA of *Rpgr* (NM_11285.2) on chromosome X was spliced in *trans* with pre-mRNA of *Eef1a1* (NM_010106.2) on chromosome 9. **(C)** The mRNA expression levels of *Rpgr*, *Eef1a1*, and *Rpgr-Eef1a1* in adult mice retina and ovary were measured by quantitative reverse transcription PCR. *β-actin* was used as an internal control. **(D)** Co-localization of RPGR-EEF1A1 and RAB37. HeLa cells were transiently co-transfected with GFP-RPGR-EEF1A1 and CHERRY-RAB37, followed by confocal microscopy. Co-localizing structures are indicated in yellow (merge). The nuclei were stained by Hoechst (blue). Scale bar, 10 μm. **(E, F)** Co-immunoprecipitation of endogenous RAB37 with FLAG-RPGR-EEF1A1 in HEK293T cells. The lysates were immunoprecipitated with anti-RAB37 (E), anti-FLAG (F) antibody, or negative serum, followed by immunoblotting with the anti-FLAG or anti-RAB37 antibody. The whole cell lysates were examined by Western blotting using anti-RAB37 or anti-FLAG antibody (Input). **(G)***In vitro* GST pull-down assay showed interaction of RAB37 with RPGR-EEF1A1. GST-RPGR-EEF1A1 and GST were incubated with HIS-RAB37 separately. Proteins pulled down with glutathione-agarose were subjected to SDS-PAGE followed by immunoblotting with an anti-RAB37 antibody. Bottom: Total proteins per lane were determined by the Pierce BCA protein assay kit. **(H)** Overexpression of RPGR-EEF1A1 promoted LC3B-II protein levels in HeLa cells (RAB37 wild-type). The promotion effect on LC3B-II levels was depleted when RAB37 was knocked down. GAPDH was used as an endogenous control. **(I)** Western blots were quantified for the LC3B-II/GAPDH ratio and the SQSTM1/GAPDH ratio. **(J, K)** Overexpression of RPGR-EEF1A1 promoted autophagosome formation in HeLa cells (RAB37 wild-type). The promotion effects were decreased when RAB37 was knocked down. LC3B-puncta were statistically analyzed in RAB37 knockdown (miR-RAB37) and control HeLa cells after starvation culture conditions (EBSS) for 1 h. Anti-LC3B and TRITC-conjugated goat anti-rabbit IgG (H + L) antibodies were used to detect LC3B-puncta (red). The nuclei were stained by Hoechst (blue). Scale bar: 10 μm. The number of LC3B dots in 20 cells was counted for each group. **(L)** RPGR-EEF1A1 interacts with RAB37 in a GTP-dependent manner. CHERRY-FLAG-RPGR-EEF1A1 was transiently co-transfected with MYC-RAB37-WT, MYC-RAB37-T43N, MYC-RAB37-Q89L, or control plasmid pcDNA 3.0 into HEK293T cells. For co-immunoprecipitation, the lysates were immunoprecipitated with anti-MYC antibody, followed by immunoblotting with anti-MYC or anti-FLAG antibodies. GAPDH was used as an internal control. **(M)** Effect of HIS-RPGR-EEF1A1 on RAB37 GTPase activity. HIS-RPGR-EEF1A1 was mixed with HIS-RAB37 by density gradient (0, 0.5, 2, and 4 μL), followed by GTPase activity assays using QuantiChromTM ATPase/GTPase kit. **(N)** HIS-RPGR-EEF1A1 was mixed with RAB37 proteins (WT and two mutants), followed by GTPase activity assays. **(O)***Rpgr* and *Eef1a1* pre-mRNAs are *trans*-spliced and generate a chimeric *Rpgr-Eef1a1* mRNA, which encodes a truncated RPGR-EEF1A1 protein. RPGR-EEF1A1 is the GEF of the small GTPase RAB37, which can activate RAB37 by stimulating GDP-to-GTP exchange and ultimately promote autophagy. ∗*P* < 0.05 and ∗∗*P* < 0.01.

Retinitis pigmentosa GTPase regulator (*RPGR*) is an X-linked gene whose mutation is one of the main causes of retinitis pigmentosa and retinal degeneration in humans and mice. More notably, our previous study has demonstrated that RPGR is a guanine nucleotide exchange factor that can stimulate RAB37 GDP-GTP exchange to promote autophagy.^3^ Hence, we explored a possible link between RPGR-EEF1A1 and the small GTPase RAB37. The interaction between RPGR-EEF1A1 and RAB37 was first investigated. Fluorescence analysis showed that RPGR-EEF1A1 and RAB37 were co-localized in the cytoplasm of HeLa cells (Fig. 1D). Further co-immunoprecipitation assays showed that RPGR-EEF1A1 could interact with RAB37 (Fig. 1E and F). GST pull-down assay revealed a direct interaction between RPGR-EEF1A1 and RAB37 (Fig. 1G; Fig. S3A). These data suggested that RPGR-EEF1A1 could interact with RAB37. As we all know, RAB37 is a small GTPase that can interact directly with ATG5 to promote autophagosome formation by regulating the assembly of the ATG5-12-16 complex.^2^ Hence, to investigate whether RPGR-EEF1A1 plays a role in autophagy via interaction with RAB37, both RAB37 wild-type and knockdown cell lines were constructed. Under starvation stress, overexpression of RPGR-EEF1A1 significantly increased the expression level of LC3B-II and significantly decreased the expression level of SQSTM1 in the RAB37 wild-type cell line. However, the effects on LC3B-II and SQSTM1 expression levels were depleted when RAB37 was knocked down (Fig. 1H and I). In addition, immunofluorescence analysis showed that overexpression of RPGR-EEF1A1 significantly promoted the formation of autophagosomes in the RAB37 wild-type cell line under starvation conditions, while the promotion effect was significantly attenuated after RAB37 knockdown (Fig. 1J and K). Thus, RPGR-EEF1A1 can promote autophagy via RAB37.

To further explore the possible regulatory relationship between RPGR-EEF1A1 and RAB37, two RAB37 mutants (RAB37-Q89L and RAB37-T43N) were constructed. Of which, RAB37-Q89L is a GTP-hydrolysis-deficient and constitutively active mutant, while RAB37-T43N is a GDP-stabilized and constitutively negative mutant. Subsequently, co-immunoprecipitation assay revealed that the RAB37-T43N mutant had a strong interaction with RPGR-EEF1A1, while the RAB37-Q89L mutant interaction with RPGR-EEF1A1 was weakly, hinting that RPGR-EEF1A1 has a function similar to that of GEF (Fig. 1L). Further examination of the effects of RPGR-EEF1A1 on GTPase activity of RAB37 showed that wild-type RAB37 had an obvious exchange between RAB37-GDP and -GTP with increasing concentration of RPGR-EEF1A1 (Fig. 1M), while the GDP-GTP exchange capacities of the two RAB37 mutants were impaired (Fig. 1N; Fig. S3B–S3E). These data suggest that RPGR-EEF1A1 has a function similar to that of GEF, by catalyzing the exchange of GDP and GTP to activate RAB37.

Overall, *trans*-splicing can occur in both eukaryotes and prokaryotes. Nonetheless, the implication, extent, and occurrence of *trans*-splicing in eukaryotes may be quite different from prokaryotes.^4^ Here, we screened a novel *trans*-spliced transcript *Rpgr-Eef1a1* in mice. The study showed that RPGR-EEF1A1 had a function similar to that of GEF, by catalyzing the exchange of GDP and GTP to activate RAB37. In mammals, there are more than 70 known RAB proteins, but most of their GEFs remain to be identified. GEF for one GTPase could interact with different GTPases through a conserved domain. For example, RPGR can interact with RAB8A as a GEF, which participates in cilia biogenesis and maintenance.^3^ *RPGR* gene encodes several isoforms sharing an amino-terminal domain homologous to the Ran GEF regulator of chromosome condensation 1 (RCC1), which can function as a GEF.^3^ Here, our research found that RPGR-EEF1A1 interacted with RAB37 and stimulated the GDP-GTP exchange, ultimately promoting autophagy (Fig. 1O). This is the first example of a *trans*-splicing molecule involved in autophagy. However, the current research on *Rpgr-Eef1a1* molecule is still at the *in vitro* stage; the functional significance of this *trans*-splicing event needs to be further confirmed *in vivo*. Therefore, in the long term, this may open new avenues for research into RNA-based therapeutic strategies. Nowadays, genetic diseases can be treated without changing the genomic sequence of the patients through reprogramming of pre-mRNAs by means of spliceosome-mediated RNA *trans*-splicing (SMaRT). As a therapeutic strategy, SMaRT can not only restore the production of normal mRNAs based on dominant negative genes, but also reduce mutant mRNA expression in animal disease models. Furthermore, an RNA exon editing therapeutic strategy, ACDN-01, which can correct the pathogenic mutations by promoting the *trans*-splicing of the synthesized RNA molecules with the endogenous pre-mRNA targets, has recently entered the phase I/II trial for the treatment of Stargardt disease.^5^ In conclusion, in the present study, we identified a novel *trans*-spliced transcript *Rpgr-Eef1a1* in mice. We also found that RPGR-EEF1A1 interacted with RAB37 and stimulated the GDP-GTP exchange, ultimately promoting autophagy. These findings may provide a potential fundamental research data for gene therapy via reprogramming of pre-mRNAs.

Although the present study provides a valuable discovery of a new *trans*-spliced transcript *Rpgr-Eef1a1*, and proposes a novel mechanism of autophagy regulation via activation of RAB37, several potential biases and limitations need to be considered. First of all, as we all know, *trans*-splicing is the process of two separate pre-mRNA molecules joining to form a chimeric RNA. This study has already employed quantitative reverse transcription PCR experiments and Hi-C analysis techniques to exclude chimeras generated by chromosomal translocation and the reverse transcription process. Nevertheless, more direct evidence, such as long-read RNA sequencing (*e.g.*, PacBio) of mouse retinal cDNA, is needed in the subsequent study. Secondly, the experiments mainly focused on overexpressing RPGR-EEF1A1 and studying its relationship with autophagy in HEK293T and HeLa cells. Although the data are informative, they may not be able to fully mimic the real physiological state. In the upcoming study, it is necessary to knock down or knock out the endogenous *Rpgr-Eef1a1* transcript in mice and use more comprehensive autophagy detection methods to investigate its effects on autophagy. Finally, inherent biases in the experimental design, such as the selection of specific cell lines, may affect the final results obtained. Future studies should validate and extend the findings by developing different animal models and utilizing primary human cells to address these limitations.

## CRediT authorship contribution statement

**Cong Li:** Writing – original draft, Methodology, Funding acquisition, Formal analysis, Data curation. **Anqi Zhu:** Formal analysis, Data curation. **Wenhui Shi:** Formal analysis, Data curation. **Yifei Wang:** Formal analysis, Data curation. **Rongjia Zhou:** Supervision. **Yanghua Tian:** Supervision, Conceptualization. **Zhimin Zhai:** Supervision, Conceptualization. **Qiang Hong:** Writing – original draft, Supervision, Funding acquisition, Conceptualization.

## Data availability

The data will be made available upon reasonable request.

## Ethics declaration

All animal experiments and methods were performed in accordance with the relevant approved guidelines and regulations, as well as under the approval of the Ethics Committee of Anhui Medical University (LLSC20220833).

## Funding

This work was supported by the 10.13039/501100001809National Natural Science Foundation of China (No. 82201804), 10.13039/501100003995Natural Science Foundation of Anhui Province, China (No. 2208085QH235), “Early Contact Research” Training Program of Anhui Medical University (China) (No. 2024-ZQKY-113), Research Fund of Anhui Institute of Translational Medicine (China) (No. 2023zhyx-B08), and National Natural Science Foundation Incubation Project of the Second Affiliated Hospital of Anhui Medical University (China) (No. 2021GQFY10).

## Conflict of interests

No potential conflict of interests was disclosed.
