# Supplementary material for: A novel chimeric RNA RPGR-EEF1A1 enhances autophagy by interaction with the small GTPase RAB37 in a GTP-dependent manner
Source: Genes Dis. 2026 Jan 31;13(6):102063. doi: 10.1016/j.gendis.2026.102063 (PMC13319670; doi:10.1016/j.gendis.2026.102063)
Supplement: Multimedia component 1 [file mmc1.docx]

Supplementary information

**A novel chimeric RNA RPGR-EEF1A1 enhances autophagy by interaction with the small GTPase RAB37 in a** **GTP-dependent manner**

Cong Li^a^, Anqi Zhu^b^, Wenhui Shi^b^, Yifei Wang^b^, Rongjia Zhou^c^, Yanghua Tian^d,^*, Zhimin Zhai^a,^*, Qiang Hong^b,^*

^a^ Department of Hematology, The Second Affiliated Hospital of Anhui Medical University, Hefei, 230601, China

^b^ School of Basic Medical Sciences, Anhui Medical University, Hefei, 230032, China

^c^ Hubei Key Laboratory of Cell Homeostasis, College of Life Sciences, Wuhan University, Wuhan, 430072, China

^d^ Department of Neurology, The Second Affiliated Hospital of Anhui Medical University, Hefei, 230601, China

* Corresponding author

*E-mail* addresses: hongqiang@ahmu.edu.cn (Qiang Hong), zzzm889@163.com (Zhimin Zhai), ayfytyh@126.com (Yanghua Tian)

**Materials and methods**

**Animals and ethics statement**

Mice (C57BL/6J) were purchased from Beijing Vital River and were raised by animal experiment center at school of Basic Medical Sciences, Anhui Medical University. Breeding conditions were at photoperiod of 12/12, room temperature of 25 °C, humidity light cycle of 55%. All animal experiments and methods were performed in accordance with the relevant approved guidelines and regulations, as well as under the approval of the Ethics Committee of Anhui Medical University.

***Trans*-splicing screening**

RNA-Seq data were collected from NCBI GEO DataSets (https://www.ncbi.nlm.nih.gov/gds/). Conserved CDS database (CCDS) were downloaded from NCBI (ftp://ftp.ncbi.nlm.nih.gov/pub/CCDS). RNA-Seq data were downloaded from NCBI Sequence Read Archive database (SRA, http://www.ncbi.nlm.nih.gov/sra). The genome sequences (mm10 for mouse) were downloaded from the public sequence database Genome Browser (http://hgdownload.cse.ucsc.edu/goldenPath/). RNA-Seq reads from each sample were first mapped to genome using BLASTn directly. To exclude the non-*trans*-spliced sequences, RNA-Seq reads mapped to two or more transcripts were then screened logically, when the read also mapped to another transcript containing the paired-end read. *Trans*-spliced transcripts were finally obtained which contained pre-mRNA regions of different genes. The chimeric RNAs were verified by RT-PCR. All primer sequences are listed in Supplementary Table S1.

**Hi-C analysis**

Hi-C data were collected from NCBI GEO DataSets (https://www.ncbi.nlm.nih.gov/gds/) with the GEO accession number GSE35519 in mouse pro-B cell lines (WT, GSM870046), including 19 autosomes and X/Y chromosomes. The genome was first divided into large bins (whole chromosomes). The total number of Hi-C sequencing reads connecting any two bins was then counted. These raw counts were normalized to correct for biases such as chromosome length and restriction site density, yielding an "observed/expected" ratio. The color intensity directly corresponds to this "observed/expected" contact frequency ratio between whole chromosomes. The relative frequencies of interactions between chromosomes were calculated as Log2 (Observed Hi-C reads/expected Hi-C reads) according to a previous study[1].

**DNA and RNA isolation and qRT-PCR**

Genomic DNA was isolated by routine methods. Total RNAs were isolated from mouse tissues by TRIzol (15596026, Thermo Fisher) according to the manufacturer’s protocol and were dissolved in 20 μL DEPC water with 0.5 μL RNasin Ribonuclease Inhibitor (Promega, WI, USA). Genomic DNA digestion was performed with 2.5 μL RQ1 RNase-free DNase (Promega, WI, USA) at 37 °C for 40 min. Reverse transcription was performed using M-MLV reverse transcriptase (Promega, WI, USA) or AMV reverse transcriptase (Promega, WI, USA), 6 μL total RNA and oligomer (dT)_18_ at 37 °C for 2 h. *β-actin* was used as internal reference gene. qRT-PCR assays were performed using SYBR Green qPCR Mix (D01010, GeneCopoeia) in a StepOne real-time PCR system (Applied Biosystems) following the routine protocol. The 2^-ΔΔCt^ method was applied to calculate the relative mRNA expression value. The specific primers can be found in Table S2.

**Plasmid constructs**

*Rpgr* CDS (NM_011285.2) was cloned into pSico-Cherry-Flag using *EcoR*I and *Xho*I to generate CHERRY-FLAG-RPGR. *Eef1a1* CDS (NM_011285.2) was cloned into pSico-BFP using *BamH*I and *Xho*I to generate BFP-EEF1A1. *Rpgr-Eef1a1* CDS was cloned into pSico-Cherry-Flag, pSico-3xFlag, pET-32a and pGEX-4T-1-GST using *EcoR*I and *Xho*I to generate CHERRY-FLAG-RPGR-EEF1A1, FLAG-RPGR-EEF1A1, HIS-RPGR-EEF1A1 and GST-RPGR-EEF1A1 fusion proteins respectively, and was cloned into pEGFP-C1 using *Xho*I and *EcoR*I to generate GFP-RPGR-EEF1A1. The plasmids MYC-RAB37, MYC-RAB37-Q89L, MYC-RAB37-T43N, CHERRY-FLAG-RAB37, HIS-RAB37, HIS-RAB37-Q89L, HIS-RAB37-T43N, miR-RAB37 and miR-lacZ were constructed according to our previous study[2]. The primers for the constructs are described in Table S3.

**Cell culture and transfection**

HEK293T and HeLa cells were cultured in DMEM (HyClone) with 10% FBS (HyClone) and penicillin/streptomycin (1%) in a 5% CO2 and 37 °C cell incubator. For transfection assays, cells were cultured in 12- or 24- well plates and Lipofectamine^TM^ 2000 (11668-019, Invitrogen) was used for transfection following the routine protocol. To establish stable RPGR-EEF1A1-expressing cell lines, HeLa cells and RAB37 knockdown (miR-RAB37) cell lines were transduced with lentivirus of FLAG-RPGR-EEF1A1. Recombinant lentiviral particles were produced by transient transfection of HEK-293T cells with packaging (pCMV-VSV-G, pMD2.G and pRSV-Rev) and recombinant (FLAG-RPGR-EEF1A1) plasmids respectively. For starvation treatments, the cells were cultured in EBSS (HyClone) for 1 h.

**Antibodies**

Primary antibodies: Anti-GAPDH antibody (CW0100, CWBio), anti-FLAG (F3165, Sigma-Aldrich), anti-LC3B (SAB4200361, Sigma-Aldrich), anti-SQSTM1 (18420-1-AP, Proteintech), anti-RAB37 (13051-1-AP, Proteintech), anti-MYC (11667149001, Roche), anti-GFP (11814460001, Roche), anti-HIS antibody (AE003, ABclonal). Secondary antibodies: HPR-goat anti-mouse IgG (H + L) antibody (31430) and HPR-goat anti-rabbit IgG (H + L) antibody (31460) were from Thermo Fisher Scientific. Goat anti-mouse IgG, light chain specific antibody (115-035-174) was purchased from Jackson ImmunoResearch Laboratories. FITC-conjugated ImmunoPure goat anti-rabbit IgG (H + L) (ZF-0311) and TRITC-conjugated ImmunoPure goat anti-rabbit IgG (H + L) (ZF-0316) antibodies were purchased from ZSGB-Bio.

**Western blot analysis**

Western blots were performed as previously described[3]. Simply, the cultured cells were collected and lysed in RIPA buffer supplemented with 1%PMSF. The acquired protein extracts were separated by 12% SDS-PAGE and then electro-transferred to polyvinylidene difluoride (PVDF) membranes. After blocking with 5% non-fat milk powder in TBST for 1 h, the membranes were incubated with primary antibodies against GAPDH (1:5000), FLAG (1:3000), LC3B (1:2000), SQSTM1 (1:2000), RAB37 (1:1000), MYC (1:2000) at 4 °C overnight. Following that, the membranes were washed by TBST and incubated with matched secondary antibody conjugated with HRP for 1 h at room temperature. Finally, bands of the membranes were visualized by an ECL detection kit (Beyotime, China) using a gel imaging detector (Bio-Rad, USA). ImageJ software was applied for protein densitometry.

**Co-immunoprecipitation assays**

Co-immunoprecipitation assays were performed according to our previous study[4]. In brief, the cells were lysed in NETN buffer consisting of 50mM Tris-HCl at pH 8.0, 0.15M NaCl, 1mM EDTA, 0.5% NP-40 and a 1x protease inhibitor cocktail (04693159001, Roche). The protein extracts were incubated with Protein G Agarose (11243233001, Roche) and specified antibody at 4 °C overnight. After centrifugation, the acquired resins were washed with NETN buffer five times. The bound proteins were eluted using loading buffer and then separated using 12% SDS-PAGE. Following that, western blots with appropriate antibodies were performed.

**Immunofluorescence analysis**

For immunofluorescence staining experiments, the cells of each group were cultured on glass coverslips. Cells were fixed with 4% paraformaldehyde at room temperature for 20 min and permeabilized with 1% Triton X-100 in PBS for 10 min. After the cells were blocked with 5% bovine serum albumin (BSA)/PBS solutions at room temperature for 30 min, the samples were incubated with anti-LC3B primary antibody overnight at 4 °C. Subsequently, the cells were incubated with TRITC-conjugated secondary antibody for 2 h at room temperature. Finally, the nuclei were stained by Hoechst. The images were captured with a fluorescence microscope (FV1000, Japan).

**Protein purification, protein concentration detection and GTPase assays**

The assays were performed according to our previously described[4]. In brief, the rosetta competent cells were transformed with HIS-RPGR-EEF1A1, HIS-RAB37-wild-type, -Q89L or -T43N respectively. The cells were cultured at 37 °C with 220 rpm and induced with 0.1mM IPTG for 24 h at 16 °C. The acquired supernatants were filtered with 0.22 μm filters, and then loaded onto Ni NTA Beads 6FF (P2010, China). After that, gradient elution with five different densities of imidazole (2, 50, 100, 200, 500 μM) were performed. Finally, to remove impurity, the pooled supernatants were dialyzed against lysis buffer (0.1M Tris/HCl, pH 7.0-8.0) and concentrated by polyethylene glycol. The amounts of recombinant proteins were detected using the Pierce BCA protein assay kit (23227, Thermo Scientific) following the manufacturer’s protocol. The RAB37GTPase activity was determined using the QuantiChromTM ATPase/GTPase kit (DATG-200, BioAssay Systems) following the manufacturer’s protocol.

**GST pull-down assays**

The assays were performed according to our previous study [4]. In brief, rosetta cells were transformed with GST, GST-RPGR-EEF1A1 and HIS-RAB37 respectively and induced by 0.1 mM IPTG for 24 h at 16 °C. The acquired supernatants were mixed with glutathione-agarose beads (P2020, China) for 4 h at 4 °C. After that, the beads were washed with NETN lysis buffer and then incubated with HIS-RAB37 supernatant at 4 °C overnight respectively. Finally, the glutathione-agarose beads were washed by NETN buffer and then separated by SDS-PAGE and western blots with the appropriate antibodies.

**Statistical analysis**

All the data were performed at least three times, and the values were presented as means ± SEM. Student’s *t*-test was used for two group statistical comparison. One-way and two-way ANOVA were performed for comparison of more than two groups. Statistical analysis was performed using GraphPad Prism 6 software. The statistically significant differences were considered when *p* value < 0.05.

**References**

1. Zhang Y, McCord RP, Ho YJ, et al. Spatial organization of the mouse genome and its role in recurrent chromosomal translocations. Cell 2012, 148:908-921.

2. Sheng Y, Song Y, Li Z, et al. RAB37 interacts directly with ATG5 and promotes autophagosome formation via regulating ATG5-12-16 complex assembly. Cell Death Differ 2018, 25:918-934.

3. Hong Q, Li C, Ying R, et al. Loss-of-function of sox3 causes follicle development retardation and reduces fecundity in zebrafish. Protein Cell 2019, 10:347-364.

4. Ying R, Li C, Li H, et al. RPGR is a guanine nucleotide exchange factor for the small GTPase RAB37 required for retinal function via autophagy regulation. Cell Rep 2024, 43:114010.

**
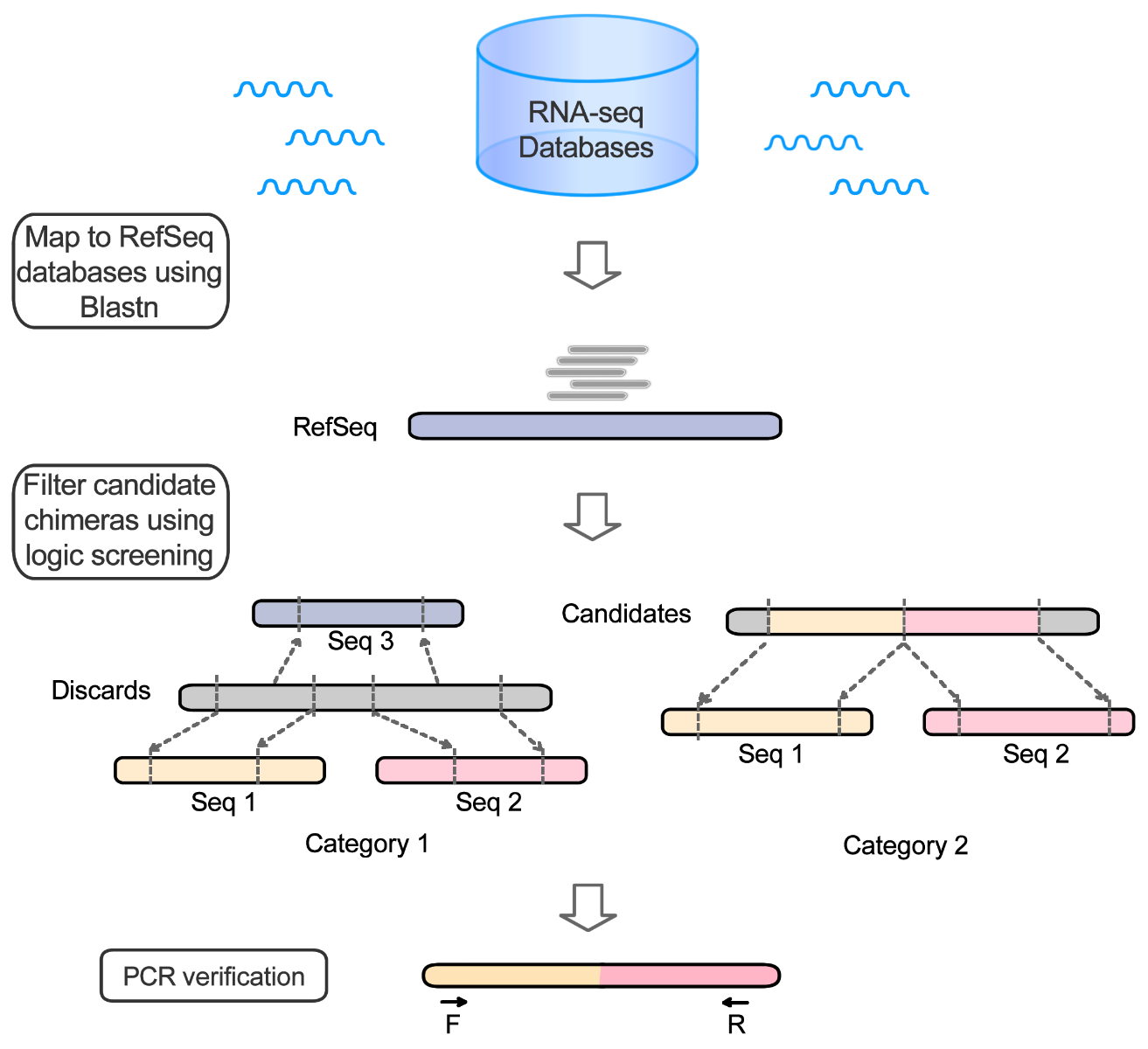
**

**Fig.S1 Screening flow chart for *trans*-splicing events.** RNA-seq reads from thirteen mouse tissues (heart: SRR4530172; liver: SRR4530150; spleen: SRR4530160; lung: SRR4530156; kidney: SRR4530144; stomach: SRR453098; duodenum: SRR4530115; colon: SRR4530166; mammary gland: SRR453091; ovary: SRR453077; testis: SRR4530143; retina: SRR4174044) were mapped to mouse RefSeq (mm10) using BLASTn, respectively. To exclude the non-*trans*-spliced sequences, RNA-Seq reads mapped to two or more transcripts were then screened logically, when the read also mapped to another transcript containing the paired-end read. *Trans*-spliced transcripts were finally obtained which contained pre-mRNA regions of different genes. The chimeric RNAs were verified by RT-PCR.

**
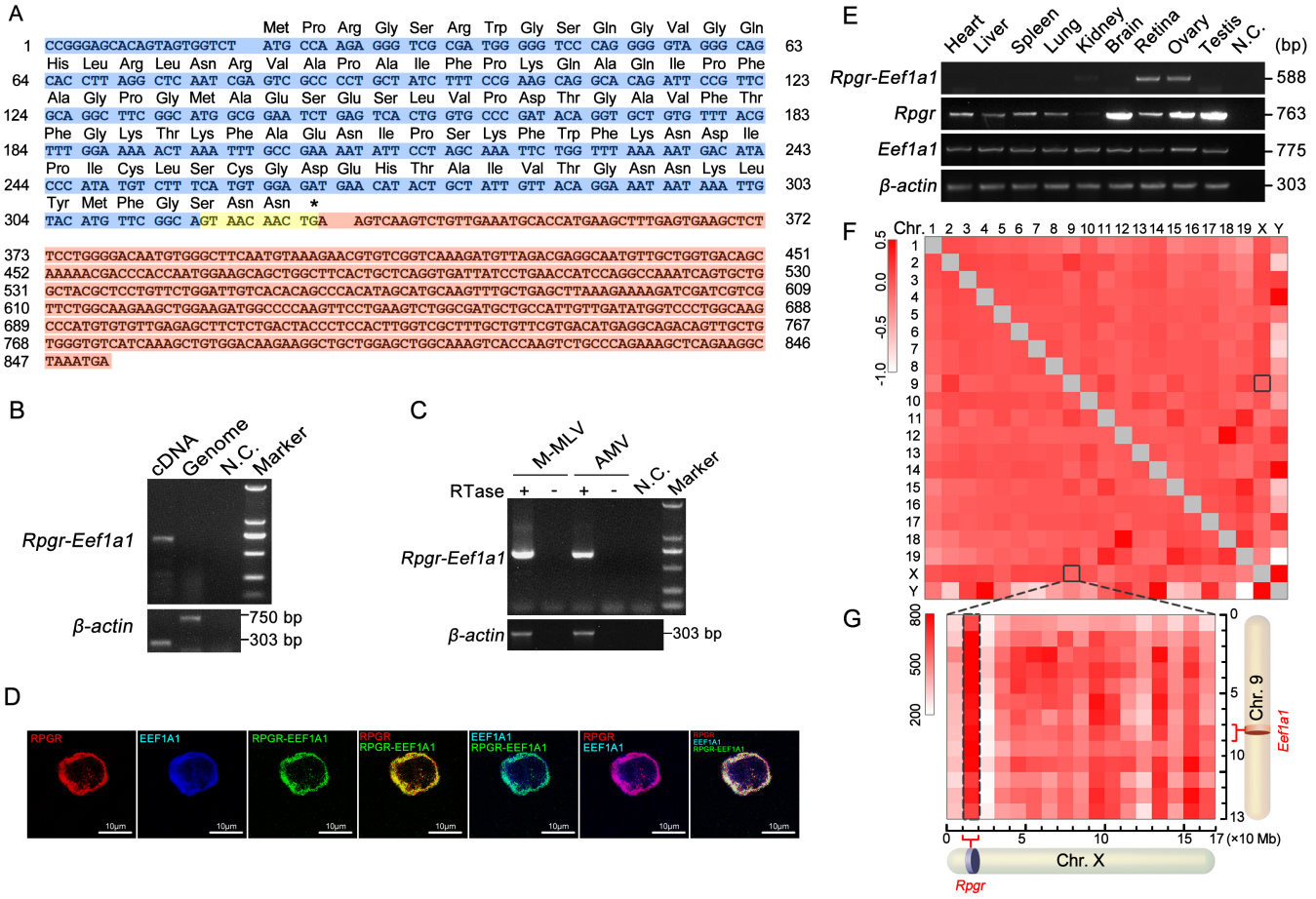
**

**Fig.S2 Identification of *trans*-spliced *Rpgr-Eef1a1* in mice.** (A) Complimentary DNA sequence of mouse *Rpgr-Eef1a1* and its coding protein. *Rpgr* (NM_011285.2) and *Eef1a1* (NM_010106.2) were marked in blue and orange. Small identical region of junction site was marked in yellow. (B) Excluding of artificial chimera. *Rpgr-Eef1a1* from genomic DNA or cDNA from retina was PCR amplified. (C) *Rpgr-Eef1a1* from cDNAs transcribed by both RTases (M-MLV and AMV) were PCR amplified. N.C.: no templet control. DNA marker: 100, 250, 500, 750, 1000 and 2000 bp. *β-actin* was used as an internal control. *β-actin*: 303 bp (cDNA) and 750 bp (genome). (D) Co-localization of *trans*-spliced RPGR-EEF1A1 and its parental RPGR and EEF1A1 proteins. HeLa cells were transiently co-transfected with GFP-RPGR-EEF1A1, CHERRY-RPGR and BFP-EEF1A1, followed by confocal microscopy. Scale bar, 10 μm. (E) The mRNA expression levels of *trans*-spliced *Rpgr-Eef1a1* and its parental genes in adult mice were analyzed by RT-PCR. *β-*actin was used as an internal control. N.C.: no templet control. (F) Hi-C analysis of spatial organization of mouse genome. Color intensity indicated observed/expected number of contacts between all pairs of whole chromosomes. Red presented enrichment and white indicated low observed/expected reads. The scale bar indicated Log2 (Observed Hi-C reads/expected Hi-C reads). (G) Heat map representing interaction map along mouse chromosome X and chromosome 9 at 10 Mb of resolution. Chromatin regions harbored *Rpgr* on chromosome X and *Eef1a1* on chromosome 9 were highlighted. The scale bar indicated read number of contacts between chromosome X and chromosome 9.


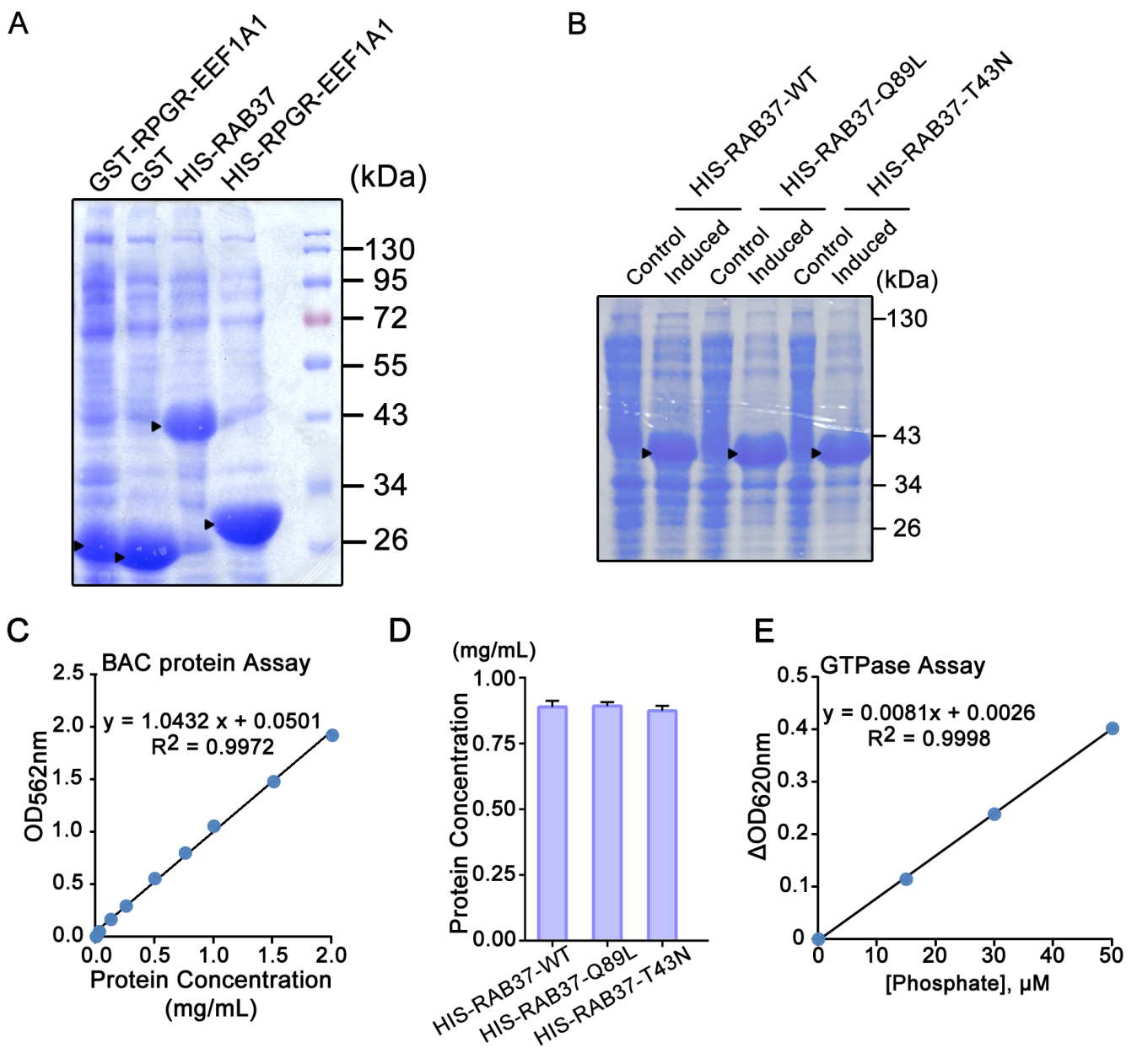


**Fig.S3 Prokaryotic induced expression.** (A) Expression of recombinant proteins for GST pull-down assays. Coomassie gel showed expression of GST-RPGR-EEF1A1, GST, HIS-RAB37 and HIS-RPGR-EEF1A1 in supernatant of *E. coli* culture. Supernatants were subjected to SDS/PAGE, and were detected by coomassie brilliant blue R250 staining. (B) Coomassie gel showed expression of HIS-RAB37-WT, HIS-RAB37-Q89L and HIS-RAB37-T43N in supernatant of *E. coli* culture. Supernatants were subjected to SDS/PAGE, and were detected by coomassie brilliant blue R250 staining. (C)The standard curve of protein concentration. (D) Protein quantification for assays of effect of RPGR-EEF1A1 on RAB37 (WT and two mutants) GTPase activity. (E) The standard curve of phosphate concentration.

| **Table S1 The primers used in the study** | | | |
| --- | --- | --- | --- |
| **No.** | **Chimeric RNA** | **Primer (5’→3’)** | **Length** |
| 1 | *Tomm5-Inpp5d* | Forward Primer：ATGTTCCGGATCGAGGGTCT  Reverse Primer：TGGTGGGGGAGTTCTGTGTT | 1073 bp |
| 2 | *E330021D16Rik-Ramp2* | Forward Primer：ATGTCGTCAGGTCTTAAGGCTG  Reverse Primer：CTGGGCATCGCTGTCTTTAC | 634 bp |
| 3 | *Rpgr-Eef1a1* | Forward Primer：ATGCCAAGAGGGTCGCGAT  Reverse Primer：TAGCCTTCTGAGCTTTCTGGG | 1244 bp |
| 4 | *Ctla2b-Pnck* | Forward Primer：ATGATGTCAGCTGCTCCATCC  Reverse Primer：ACCACTTGGGGGACTGGCTA | 678 bp |
| 5 | *Anln-Inha* | Forward Primer：ATGGACCCGTTTACCGAGAA  Reverse Primer：CTCCATCTGAGGTGGTTCGG | 1712 bp |
| 6 | *Arfgap1-Hsd3b4* | Forward Primer：AGCCCAAGAACCAGGAAAGTT  Reverse Primer：TGTCTCCCTGTGCTGCTTCAC | 1126 bp |
| 7 | *Lingo1-Hspa2* | Forward Primer：ATGCTGGCAGGGGGTATGA  Reverse Primer：GTCCACTTCCTCGATGGTGG | 1570 bp |
| 8 | *Phlda1-Cd37* | Forward Primer：CCTCTCGGAGCTGGGTTTT  Reverse Primer：GCCGGTCATAGACGTGATCC | 872 bp |
| 9 | *Snrpn-Emilin1* | Forward Primer：ATCCTGCAAGATGGGAGAATC  Reverse Primer：GCTCCGCTGAAGATGGTGA | 927 bp |
| 10 | *Gfod1-Yy1* | Forward Primer：GCCTCACAGCCCGAGTCAT  Reverse Primer：TCACTGGTTGTTTTTGGCTTTA | 1208 bp |
| 11 | *Kcnq2-Pdlim4* | Forward Primer：CACCAGCGGGGAAAAGAA  Reverse Primer：CAGACAAGTTCCACCTTAGCATT | 1003 bp |
| 12 | *Ppm1b-Ctsd* | Forward Primer：ATGGGTGCATTTTTGGATAAAC  Reverse Primer：TTAGAGTACGACAGCATTGGCA | 701bp |
| 13 | *Vegfa-Igfbp7* | Forward Primer：CTGACGGACAGACAGACAGACA  Reverse Primer：CTGAGCACCTTCACCTTTTTTC | 860 bp |
| 14 | *Olig1-Msc* | Forward Primer：ATGTACTATGCGATTTCCCAGG  Reverse Primer：TAAGCGGAAGTTCCACAAAGC | 609 bp |
| 15 | *Angptl2-Rhou* | Forward Primer：ATGAGGCCACTGTGTATGACCT  Reverse Primer：TCAGGCCAGGCAGCAATACT | 1118 bp |
| 16 | *Ppard-Esrrg* | Forward Primer：ATGAGAAGTGCGATCGGATCT  Reverse Primer：TTCATCCTCAAACGAAAGCGA | 503 bp |

| **Table S2.** **The primers used in the study** | | |
| --- | --- | --- |
| **Genes/fragments** | **Primer sequence (5’ - 3’)** | Tm (°C) |
| *Rpgr-Eef1a1* | F: CCTGCTATCTTTCCGAAGCAG  R: ATATCAACAATGGCAGCATCG | 58 |
| *β-actin* (NM_007393.5) | F: GTGGGAATGGGTCAGAAGGA  R: TGCTGTCCCTGTATGCCTCT | 58 |
| *Rpgr* (NM_11285.2) | F: ATGCCAAGAGGGTCGCGATG  R: GTTCTCCAAATGTGTAGAGC | 58 |
| *Eef1a1* (NM_010106.2) | F: TGCTAATATGCCTTGGTTCA  R: TCATTTAGCCTTCTGAGCTTTC | 58 |

| **Table S3. The plasmids constructed in the study** | | |
| --- | --- | --- |
| **Plasmid name** | **Primer name** | **Primer sequence (5’ - 3’)** |
| CHERRY-FLAG-RPGR | Rpgr-*EcoR* I-F  Rpgr-*Xho* I-R | CGGAATTCATGCCAAGAGGGTCGCGAT  CCGCTCGAGTTATAGAATTGTACAGGATTT |
| BFP-EEF1A1 | BFP-Eef1a1-*BamH* I-F  BFP-Eef1a1-*Xho* I-R | CGGGATCCATGGGAAAGGAAAAGACTCAC  CCGCTCGAGTCATTTAGCCTTCTGAGCTTT |
| GFP-RPGR-EEF1A1 | GFP-R-E-*Xho* I-F  GFP-R-E-*EcoR* I-R | CCGCTCGAGCTATGCCAAGAGGGTCGCGAT  CCGGAATTCTTATAGAATTGTACAGGATTT |
| FLAG-RPGR-EEF1A1 | R-E-*EcoR* I-F  R-E-*Xho* I-R | CGGAATTCATGCCAAGAGGGTCGCGAT  CCGCTCGAGTCAGTTGTTACTGCCGAACA |
| GST-RPGR-EEF1A1 | R-E-*EcoR* I-F  R-E-*Xho* I-R | CGGAATTCATGCCAAGAGGGTCGCGAT  CCGCTCGAGTCAGTTGTTACTGCCGAACA |
| HIS-RPGR-EEF1A1 | R-E-*EcoR* I-F  R-E-*Xho* I-R | CGGAATTCATGCCAAGAGGGTCGCGAT  CCGCTCGAGTCAGTTGTTACTGCCGAACA |
| CHERRY-FLAG-RPGR-EEF1A1 | R-E-*EcoR* I-F  R-E-*Xho* I-R | CGGAATTCATGCCAAGAGGGTCGCGAT  CCGCTCGAGTCAGTTGTTACTGCCGAACA |
| ***** Sites for restriction enzymes are underlined. PCR condition: 94 °C, 30 s; 65°C -1°C, 30 s; 70°C, 2 min, 10 cycles; 94 °C, 30 s; 60°C, 30 s; 70°C, 2 min, 25 cycles | | |
